# Predicting therapeutic drugs for hepatocellular carcinoma based on tissue-specific pathways

**DOI:** 10.1371/journal.pcbi.1008696

**Published:** 2021-02-09

**Authors:** Liang Yu, Meng Wang, Yang Yang, Fengdan Xu, Xu Zhang, Fei Xie, Lin Gao, Xiangzhi Li

**Affiliations:** 1 School of Computer Science and Technology, Xidian University, Shaanxi, China; 2 Shandong Provincial Key Laboratory of Animal Cell and Developmental Biology, School of Life Sciences, Advanced Medical Research Institute, Shandong University, 72, Jimo District, Qingdao, Shandong, China; University at Buffalo - The State University of New York, UNITED STATES

## Abstract

Hepatocellular carcinoma (HCC) is a significant health problem worldwide with poor prognosis. Drug repositioning represents a profitable strategy to accelerate drug discovery in the treatment of HCC. In this study, we developed a new approach for predicting therapeutic drugs for HCC based on tissue-specific pathways and identified three newly predicted drugs that are likely to be therapeutic drugs for the treatment of HCC. We validated these predicted drugs by analyzing their overlapping drug indications reported in PubMed literature. By using the cancer cell line data in the database, we constructed a Connectivity Map (CMap) profile similarity analysis and KEGG enrichment analysis on their related genes. By experimental validation, we found securinine and ajmaline significantly inhibited cell viability of HCC cells and induced apoptosis. Among them, securinine has lower toxicity to normal liver cell line, which is worthy of further research. Our results suggested that the proposed approach was effective and accurate for discovering novel therapeutic options for HCC. This method also could be used to indicate unmarked drug-disease associations in the Comparative Toxicogenomics Database. Meanwhile, our method could also be applied to predict the potential drugs for other types of tumors by changing the database.

## Author summary

Selecting therapeutic drugs that can be used for cancer treatment from existing drugs is a way to quickly obtain effective drugs, whereas the safety, dose and marginal effects of these drugs have been verified, and their clinical application will be faster than that of novel drug discovery. There have been a large number of successful cases of drug repositioning in the past few years. Tumors have complex regulatory pathways; thus, with the help of the HCC regulatory pathway, the single gene expression changes in the pathway, and the specific correlation between HCC and liver tissue, we screened for the predicted drugs, and constructed Connectivity Map and Toxicogenomics Database combined with KEGG pathway to carry out these drugs. Also, we verified the therapeutic effect of selected drugs *in vitro* to confirm the effectiveness of these drugs. Our results show that our drug screening method is effective, and can be applied to other malignant tumor drug screening.

## Introduction

Hepatocellular carcinoma (HCC), also called malignant hepatoma, is a major health problem and one of the leading causes of death worldwide, with an annual number of cases exceeding 841,000 [1]. HCC mostly affects patients with liver cirrhosis, which is the most common reason for the death of these patients. Only a few safe and effective therapeutic options are available to HCC patients, however, and the current drug discovery process is both costly and time inefficient [2]. By a rough estimation, it can take up to 15 years [3,4] and $ 2.8 billion to bring a single drug to the market [4]. In response to this slow process, drug repositioning has been used as a profitable and successful strategy for drug discovery and development. Because existing drugs usually are vetted in terms of safety, dosage, and toxicity, they can be applied in clinical phases more rapidly than newly developed drugs [5]. Well-known examples include minoxidil (originally designed for hypertension now used to treat hair loss) [6], sildenafil (originally designed for pulmonary hypertension now used to treat erectile dysfunction) [7], amphotericin B (originally designed for serious systemic fungal infections now used to treat acute promyelocytic leukemia) [8], and atorvastatin (originally designed for cardiovascular disease now used to treat Alzheimer’s disease) [9].

Many computational methods for new drug indications using various strategies were proposed. Human genetics studies have offered the strongest evidence thus far to connect genes to human diseases. In 2016, Zhang et al. [10] identified 22 liver cancer–related enhancer single-nucleotide polymorphisms by integrating genome-wide association study (GWAS) data and histone modification ChIP-seq data. Sanseau and Agarwal [11] found that disease genes extracted from GWAS data were 2.7-fold more likely to be drug targets. In addition, they uncovered 123 drug targets associated with GWAS traits that were not related to their original indications. These mismatches opened the door for drug repositioning. Bamshad et al. [12] dissected the genetic basis of diseases and traits by exome sequencing. Their strategies have focused on identifying individual genes that exhibit differences in expression between normal and disease states. In fact, gene products within a cell do not function alone [13]. They interact with each other to carry out specific biological functions. Complex diseases generally are caused by the dysregulation of a set of genes that share a common biological function [13–15].

Pathway-based computational approaches have been proposed recently to overcome problems that consider only a single-gene function [15]. Ye et al. [14] integrated known drug target information and proposed a disease-oriented strategy to evaluate the relationship between drugs and diseases based on the pathway profiles of diseases. Pathway profile is a vector with 185 dimensions and each element in the vector is the *p*-value of the hypergeometric test corresponding to 185 Kyoto Encyclopedia of Genes and Genomes (KEGG) pathways (https://www.genome.jp/kegg/) [16]. Later, Li et al. [17] constructed a multilayer causal network (CauseNet) consisting of chains from drug to target, target to pathway, pathway to downstream gene, and gene to disease. The transition likelihood of each causal chain in the network was measured by a statistical method based on known drug–disease treatment relationships. Yu et al. [18–22] proposed computational methods to study drug repositioning based on different network models, such as module distance [18], protein complexes [19], and triangularly balanced structure [22]. Zhao et al. [21] extended the concept of cancer signaling bridges and developed a computational model to derive specific downstream signaling pathways that revealed previously unknown target-disease connections and new mechanisms for specific cancer subtypes. Pathway analysis can effectively uncover the perturbation of biological systems by diseases [23]. A common problem with these methods, however, is that they treated pathways simply as gene sets and ignored the changes of a single gene in pathways and tissue-specific information of the pathways [14,16,19].

In this study, we proposed a novel approach to identify related drugs for the treatment of HCC, which considers the functional information of pathways, the changes of a single gene in pathways, and the special relationship between HCC and liver tissue. We searched for that up-regulated and down-regulated genes in each cancer-associated pathway that was counter-regulated the most by each drug instance in the Connectivity Map (CMap) [24] database. The degree of counter-regulation of those two kinds of genes was combined for each drug instance. The combined degrees were averaged over all cancer-associated pathway genes and instances of each drug, giving one therapeutic score for each drug. Drugs were ranked by the therapeutic scores, and top ranked drugs selected for further investigation. We validated these predicted drugs by analyzing their overlapping drug indications reported in PubMed literature. By using the cancer cell line data in the database, we constructed a Connectivity Map (CMap) [24] profile similarity analysis and KEGG enrichment analysis on their related genes. These results suggested that our method could clearly distinguish among the potential drugs that are therapeutic for HCC, which is helpful when annotating positive or negative drug–disease associations in the Comparative Toxicogenomics Database (CTD) (http://ctdbase.org/) [25]. Furthermore, the effect of drugs on the expression of HCC-associated genes were verified *in vitro*. The results showed that securinine, one of our predicted drugs, exhibited significant cytostatic effects on HCC cells but not on normal cells. At the same time, another drug, ajmaline, has also been shown to have a significant cytotoxic effect on HCC cell lines. These findings suggested that the approach proposed in this study is effective for accurate discovering novel therapeutic options for HCC. Furthermore, the method of data analysis in this study could also be used to predict the potential drugs for other types of tumors by changing the database.

## Results

### Analysis of HCC gene expression data, drug-related gene expression data and tissue-specific subnetwork of HCC

#### HCC gene expression data

We downloaded the HCC gene expression profiles from the National Center for Biotechnology Information Gene Expression Omnibus (GEO, http://www.ncbi.nlm.nih.gov/geo/) [26]. The gene expression data set (GSE45436) contains 134 samples, including 95 tumor samples and 39 control samples [27]. We mapped each probe to a gene using the GPL570 platform obtained from GEO and removed the probes if they did not match any gene. If multiple microarray probes were mapped to the same gene, we averaged the expression values. Here, we normalized the expression values for all genes. The details are given in the Methods section.

#### Gene expression data related to drugs

We downloaded gene expression data related to drugs from the CMap (http://www.broadinstitute.org/cmap/) database [24]. The database contains 6,100 instances, covering 1,309 drugs. We measured these instances in five types of human cancer cell lines, including breast cancer epithelial cell lines MCF7 and ssMCF7, prostate cancer epithelial cell line PC3, nonepithelial lines HL60 (leukemia), and SKMEL5 (melanoma).

#### Tissue-specific subnetwork of HCC

**Fig 1:**
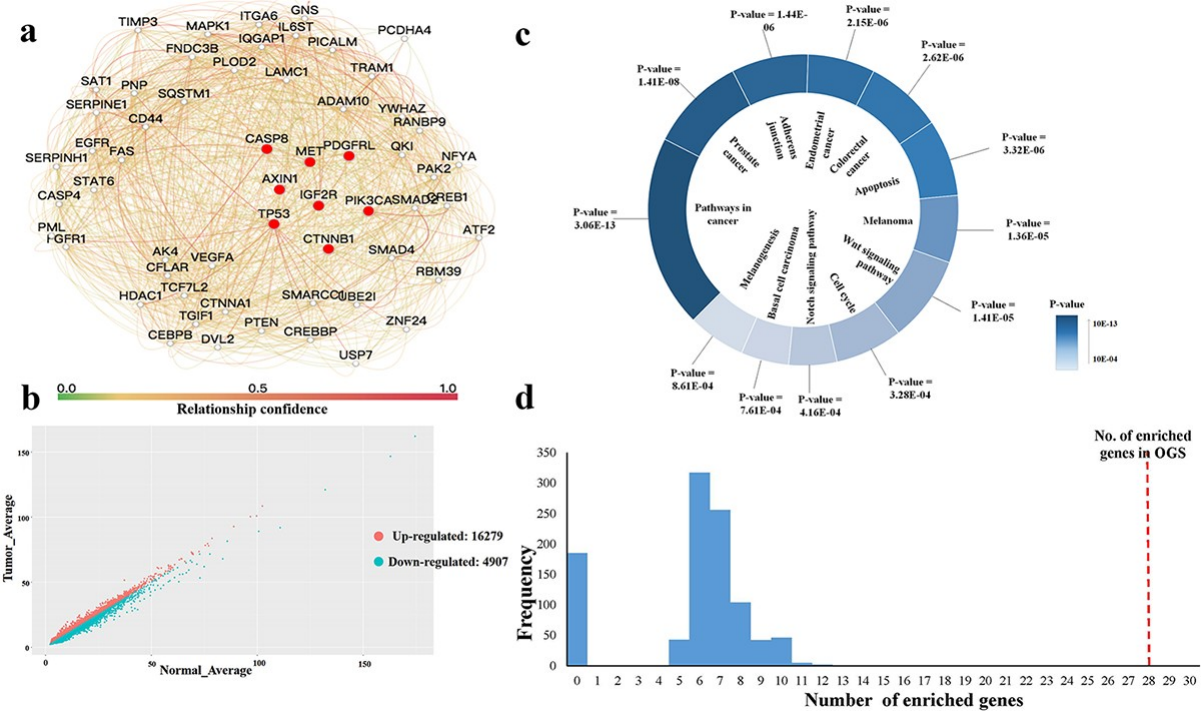
The HCC-specific subnetwork. **(a)** The subnetwork is extracted from the liver PPI network, which includes 57 genes and 838 edges. The genes related to HCC obtained from OMIM are marked by a red circle. **(b)** The distribution of up- and downregulated genes related to HCC. **(c)** Twelve HCC-specific pathways with lower *p*-values. The *p*-values represents the number of HCC-specific genes enriched in the corresponding KEGG pathway. **(d)** The significance of the OGS of HCC is verified by comparing them with 1,000 random gene sets. The x-axis represents the number of genes involved in 12 HCC-specific KEGG pathways for a random gene set. The y-axis represents the frequency of the 10,000 random gene sets with the corresponding number of enriched genes. The red-dotted line in the right represents the 28 genes in the original gene set enriched in 12 HCC-specific KEGG pathways. OGS: Original Gene Set.

To obtain the tissue-specific subnetwork of HCC, we identified eight genes associated with HCC from Online Mendelian Inheritance in Man (OMIM, http://www.omim.org) [28,29], shown in Table 1. MET, PIK3CA, and CTNNB1 genes play a critical promoting role in the origin of HCC [30–32]. Other genes are important tumor suppressor genes, and loss of function mutations for these genes may lead to the development of HCC [33–37]. Using these eight genes as seeds, we extracted a subnetwork by including their direct neighbors in the liver-specific protein–protein interaction (PPI) network in the Genome-Scale Integrated Analysis of Networks in Tissues (GIANT) (http://giant.princeton.edu/) [38]. GIANT is a dynamic, interactive web interface of tissue-specific networks. We set the cutoff of relationship confidence at 0.1. The final HCC-specific subnetwork contained 57 genes and 838 edges, shown in Fig 1A and S2 Table. This subnetwork was characterized by high average clustering coefficient, high node degree, and small average shortest path length (Table 2). In other words, the subnetwork was dense and showed strong connections between HCC genes and other genes. We named the 57 genes in the subnetwork as the HCC original gene set (OGS).

**Table 1:** HCC-related genes extracted from OMIM.

****Gene Symbol****	****Entrez Gene ID****	****Role****
IGF2R	3482	S
CASP8	841	S
MET	4233	O
PDGFRL	5157	S
TP53	7157	S
PIK3CA	5290	O
CTNNB1	1499	O
AXIN1	8312	S

Genes are classified into two types: oncogene (Role = "O") and tumor suppressor genes (Role = "S").

**Table 2:** Topological attributes of the HCC-specific subnetwork.

****Attribute name****	****Value****
Number of nodes	57
Number of edges	838
Average node degree	28.9
Average clustering coefficient	0.69
Average shortest path length	1.5
Maximal diameter	3

### Identification of gene expression profiles of HCC

By analyzing the gene expression data of HCC, we obtained a ranked gene list of HCC based on their expression values. We divided genes in the list into two sets: the up- and downregulated sets (distribution is shown in Fig 1B). Before division, genes were ranked by decreasing logFC [39,40], actually logFC = log_2_(mean(*tumor*)/mean(*control*)), where mean(*tumor*) and mean(*control*) represent the average expression values of a gene across all tumor samples and all control samples, respectively. If the logFC of a gene was greater than zero, then the gene was upregulated; otherwise, it was downregulated.

### Detection of HCC tissue-specific pathway signatures

HCC is a complex disease and complex diseases generally involve several gene mutations and pathway dysregulations [41,42], we further analyzed the biological functions of these 58 genes in the tissue-specific PPI network of HCC. Using the database for annotation, visualization, and integrated discovery (DAVID) tool (https://david.ncifcrf.gov/) [43,44], we performed pathway enrichment analysis on these genes. We set the parameters of DAVID as follows: *p*-value = 0.001 and count = 5. We obtained 12 KEGG pathways [16] (Fig 1C), together containing 28 of 57 (49.1%) HCC-specific genes. We named these 12 KEGG pathways as HCC-specific pathways. Then, based on the ranked gene list of HCC based on their expression values (see section “Identification of gene expression profiles of HCC”), we ranked the genes in each HCC-specific pathway in Fig 1C and obtained 12 ranked HCC-specific gene lists, which we named as HCC tissue-specific pathway signatures (see S3 Table for details).

### Verification of the OGS signature

It was necessary to retain the eight seed genes; thus, we randomly selected only 49 genes from the liver PPI networks. We performed pathway enrichment analysis on the random gene set using the DAVID tool with the same parameter. We counted the number of genes that were enriched in the KEGG pathway and compared it with that of OGS. We repeated this procedure 1,000 times. None of these pathways produced more than 28 genes (shown in Fig 1D), which suggested that the OGS of HCC were significantly functionally related.

### Calculation of the therapeutic scores of drugs

**Fig 2:**
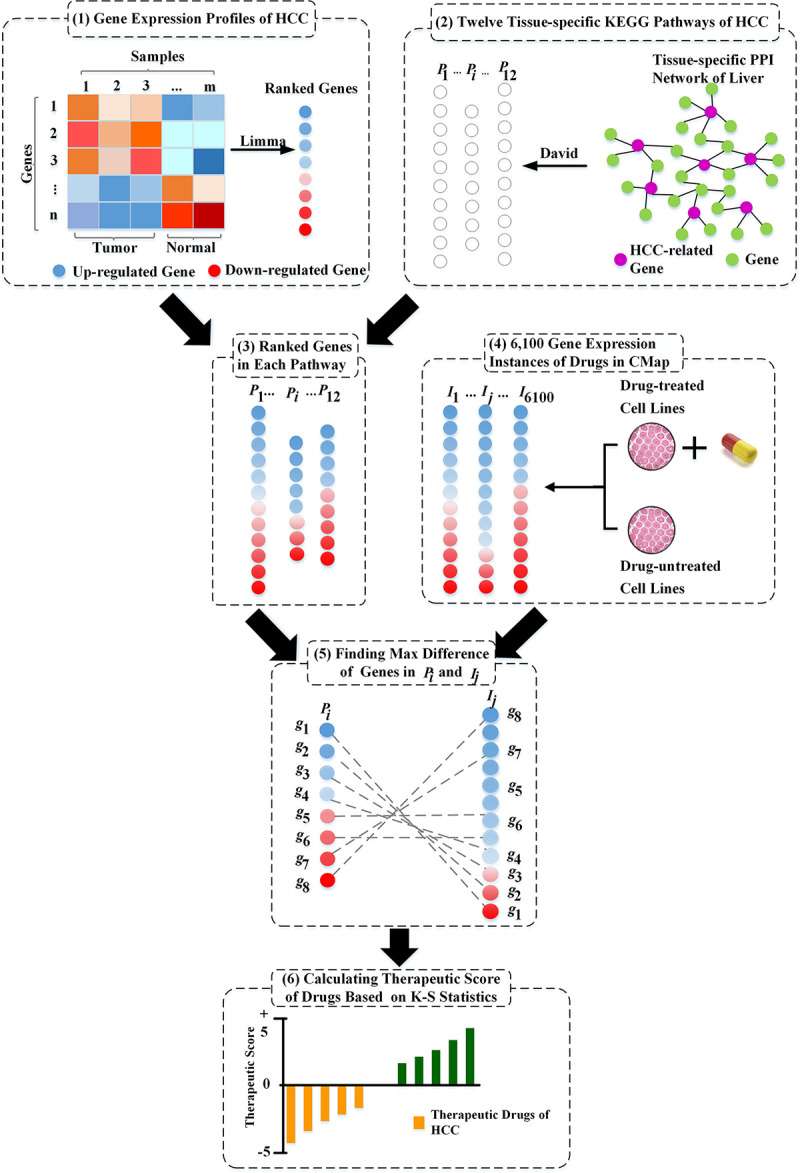
The framework of the method to predict related drugs for HCC. (1) Control samples were compared with tumor samples using Limma [46] to generate a list of upregulated and downregulated genes for HCC disease. Genes were ranked by their expression values. The red circles represent upregulated genes, and the blue circles represent downregulated genes. (2) Disease-related genes and liver tissue–specific network were integrated to construct a tissue-specific gene signature of HCC. Based on the tissue-specific gene sets, 12 related pathways were extracted using KEGG pathway enrichment analysis. The purple circles represent the genes related to HCC, and the other green circles represent other genes in the network. (3) Genes in each pathway are ranked based on their expressions obtained from (1). (4) A total of 6,100 gene expression instances of drugs got from the Connectivity Map. (5) Different orders of the same gene in the pathway *P*_*i*_(*i* = 1,2,…,12) and instance *I*_*j*_(*j* = 1,2,…,6100). On the basis of the different orders of genes in pathway *P*_*i*_(*i* = 1,2,…,12) and instance *I*_*j*_(*j* = 1,2,…,6100), we calculated the therapeutic score of each drug based on the Kolmogorov–Smirnov test [24,45]. (6) Drugs are ranked according to their therapeutic scores. The orange bars (therapeutic scores < 0) represent the therapeutic drugs.

In this section, we focused on the pathway genes with the largest expression difference in HCC and drug DEGs (Differentially Expressed Genes) to reverse pathway activities by targeting the most promising candidate. We obtained 6,100 instances of gene expression covering 1,309 drugs from the CMap database [24]. In other words, a drug may correspond to multiple instances of gene expression. We ranked the genes in each instance by taking the differential expression values between drug-treated and drug-untreated cell lines (shown in Fig 2 (4)) and generated 6,100 drug-related gene lists. On the basis of the 12 HCC-specific pathways and 6,100 instances of drug-related gene expression, we used a nonparametric, rank-based pattern matching strategy Kolmogorov–Smirnov test [45], originally introduced by Lamb et al. [24], to evaluate the relationship between drugs and HCC (shown in Fig 2 (5)).

**Fig 3:**
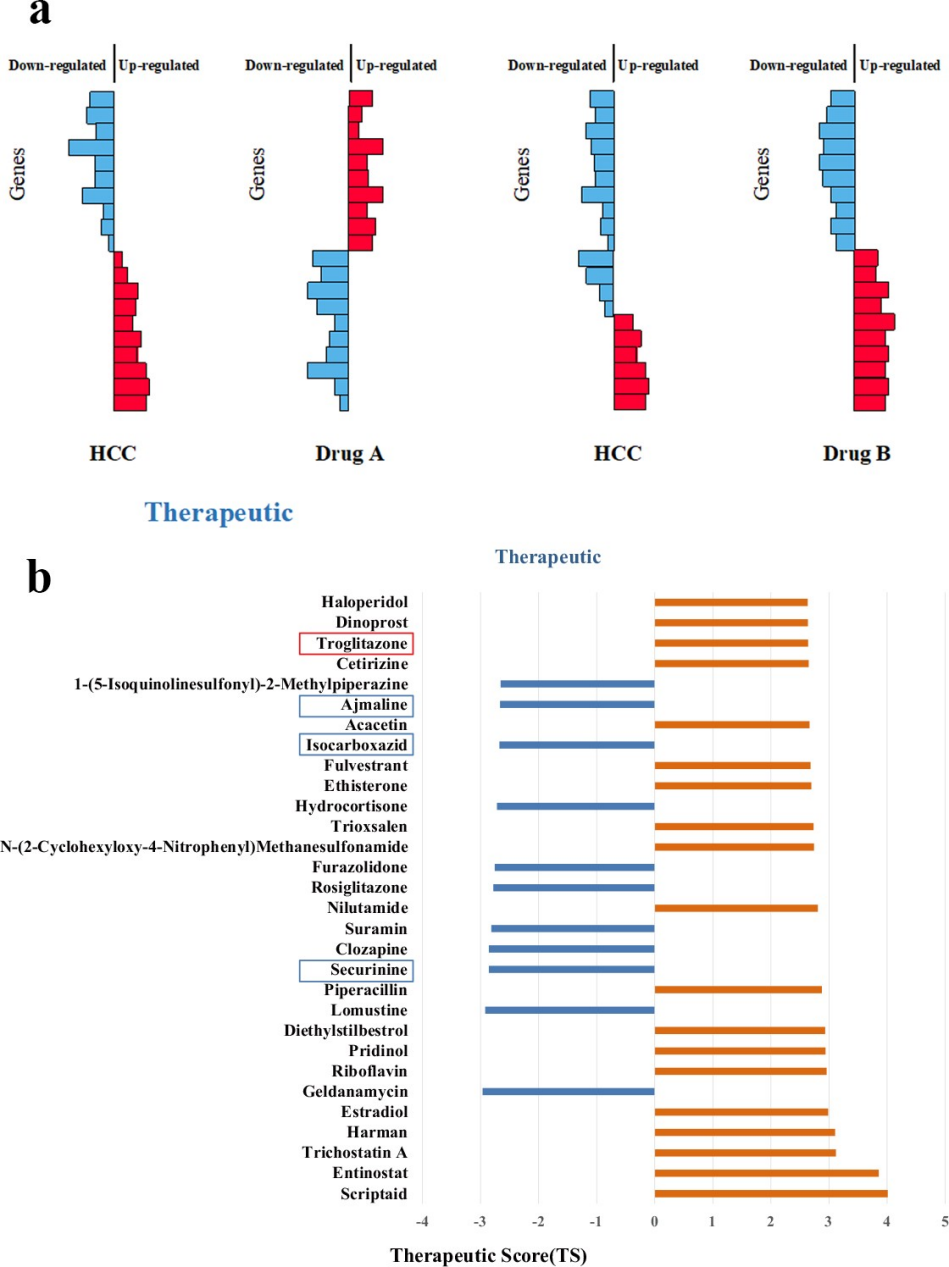
The relation of gene expression level between disease and drug. **(a)** When the gene expression profiles are reversed, the drug is likely to treat the disease. **(b)** The distribution of therapeutic scores of top-30 drugs. There are 12 therapeutic (blue bars) for HCC. The drug “Troglitazone” marked in the red box is the drug marked as “Therapeutic” in the CTD database. The three drugs marked in the blue box, securinine, isocarboxazid, and ajmaline, are our newly predicted drugs, which are not recorded in the CTD. The rest of the drugs are marked as "inferred" in the CTD database.

We took each ranked drug expression profile as the reference signature and assessed their similarity, named as the Therapeutic Score (*TS*), with each HCC pathway (see the details in Methods section). As shown in Fig 3A, if the upregulated pathway genes were near the bottom of the rank-ordered drug gene lists and the downregulated pathway genes were near the top of the rank-ordered drug gene lists, we obtained negative *TS* values. This result implied that the given drugs and the HCC pathways had adverse expression profiles and that the drugs could be candidates for HCC treatment. Conversely, if the upregulated pathway genes were near the top (upregulated) of the rank-ordered drug gene lists and the downregulated pathway genes were near the bottom (downregulated) of the rank-ordered drug gene lists, we obtained high positive *TS* values.

### Selection of potential HCC drugs through CTD benchmark

To find the most likely HCC drugs, we evaluated the precision of our first method. We took CTD [25] as the benchmark, which provided manually curated information about drug–gene interactions, drug–disease relationships, and gene–disease relationships. We extracted curated chemical–disease associations from the published literature by CTD biocurators and established inferred associations by CTD-curated chemical–gene interactions.

If the corresponding chemical name of a drug in CMap cannot be identified in CTD, we cannot calculate its therapeutic score (see the details in Methods section). In this way, we obtained 1,168 scored drugs. Because most drug–disease associations in CTD are not marked as positive or negative, we ranked the 1,168 drugs in descending order using the absolute values of their therapeutic scores (*TS*) (S4 Table). As we know, the top drugs indicate stronger connections with HCC. We then calculated the precision of our approach at different top-*x* drugs. *Precision* was calculated as follows:

$$\mathrm{precision}=\frac{P_{\mathrm{CTD}}}{P}$$ (1)

Where *P* represents the number of top-*x* drugs; *P*_CTD_ represents the number of drugs in the top-*x* drugs can be found related to HCC in CTD database.

In the top-10 drugs (*x* = 10), we found nine drugs associated with HCC in CTD (i.e., precision = 0.9). For the top-20 drugs (*x* = 20), the precision was 0.85, and we identified seventeen drugs associated with HCC in CTD and the other three drugs that potentially were HCC related. When *x* was 30, its precision was 0.8, and we obtained twenty-four drugs associated with HCC in CTD and the other six drugs were potentially related to HCC. We noticed that with an increase of *x*, the precision decreased and the number of potential drugs increased. We hoped to predict relatively more HCC-related drugs with high precision. Therefore, we selected top-30 (*x* = 30) drugs for further analysis. The distribution of their therapeutic scores is shown in Fig 3B. From Fig 3B, we find 12 therapeutic (blue bars) drugs for HCC. In the following section, we analyze these drugs further.

### Validation of potentially HCC-related drugs through PubMed search

In the previous section, we selected the top-30 drugs (precision = 0.8) for further analysis. We identified 12 therapeutic drugs with negative *TS* values. Nine of these drugs had a connection with HCC in CTD. But only one in nine drugs was marked as “therapeutic” (Rank = 12 and Evidence = "T" shown in Table 3). The other eight inferred drugs were not marked in CTD. Our results showed that these eight unmarked drugs were more likely to be therapeutic drugs for HCC. The remaining three drugs, securinine, isocarboxazid, and ajmaline (in bold in Table 3), were newly predicted using our method.

**Fig 4:**
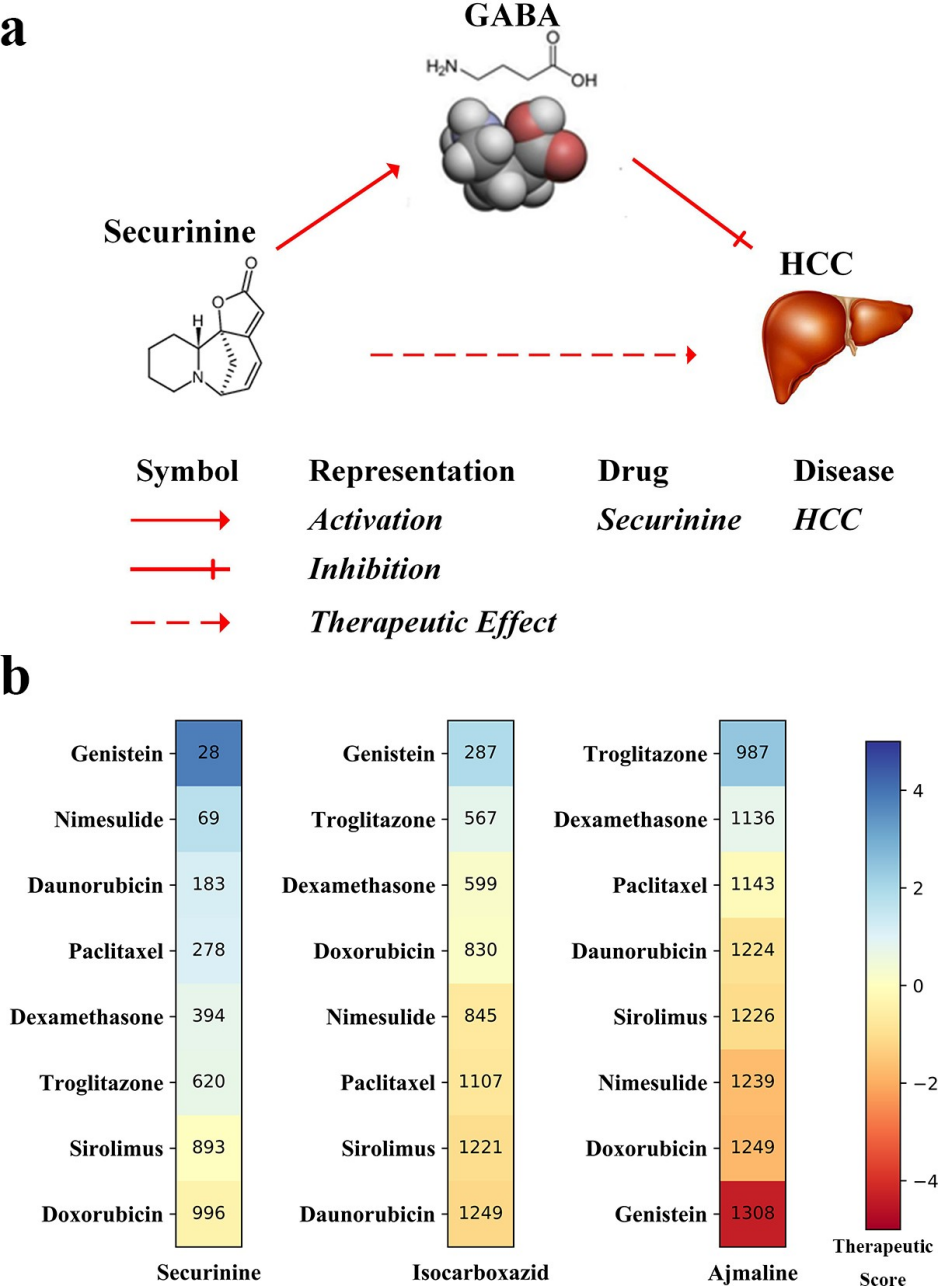
Description of the mechanism of drugs and HCC. **(a)** The mechanism of securinine treating HCC. Securinine has been found to be active as a γ-amino butyric acid (GABA) receptor antagonist. GABA stimulates HCC cell line HepG2 growth. Consequently, securinine is a promising agent with a therapeutic effect on HCC patients by inhibiting GABA receptor. **(b)** The relationships (Therapeutic Scores) among predicted therapeutic drugs, securinine, isocarboxazid, and ajmaline with eight HCC therapeutic drugs based on their related gene expression data obtained from CMap. The numbers in the boxes represent the ranks of the correlations between the three selected drugs securinine, isocarboxazid and ajmaline and the eight therapeutic drugs of HCC (shown on the y-axis).

- Securinine (Rank = 3 in Table 3), a quinolizine pseudoalkaloid (not from the amino acid) from *Securinega suffruticosa* Rehd, is a central nervous stimulant and clinically applied to treat amyotrophic lateral sclerosis, poliomyelitis, and multiple sclerosis [47–49]. It has been found to be active as a γ-amino butyric acid (GABA) receptor antagonist [50]. GABA is the main inhibitory neurotransmitter in the central nervous system and plays a major role in reducing neuronal excitability throughout the nervous system. Studies have shown that GABA stimulates HCC cell line HepG2 growth [51]. These findings suggest that securinine is a promising agent with the therapeutic potential for HCC by inhibiting GABA receptor. The mechanism is depicted in Fig 4A.
- Isocarboxazid (Rank = 9 in Table 3) is a nonselective, irreversible monoamine oxidase inhibitor (MAOI) of the hydrazine class used as an antidepressant [52]. Studies have shown that up-regulation of MAO expression in tumors can lead to increased tolerance of cells to chemotherapy [53]. As a nonselective MAOI, isocarboxazid may be used as a separate chemotherapy drug or as an adjuvant chemotherapy drug to enhance the sensitivity of tumors to chemotherapy drugs.
- Ajmaline (Rank = 10 in Table 3) is a sodium ion channel blocker. It serves as a treatment for Wolff–Parkinson–White syndrome, which is a disorder of the electrical system of the heart referred to arrhythmia with the ventricles contracting prematurely. Sodium channel blockers are drugs that impair conduction of sodium ions (Na^+^) through sodium channels. The sustained low extracellular sodium ion concentration decreases glutamate uptake [54]. Glutamine is an essential compound in cellular metabolism and a fuel for cell proliferation, and the decrease of glutamine will inhibit tumor proliferation [55]. Hence, ajmaline provides an accessible therapeutic window for HCC treatment.

**Table 3:** Twelve therapeutic drugs for HCC in the top-30 drugs.

****Rank****	****Drug Name****	****Evidence****	****Ref Count****
1	Geldanamycin	Inferred	33
2	Lomustine	Inferred	1
****3****	****Securinine****	****NA****	****NA****
4	Clozapine	Inferred	35
5	Suramin	Inferred	14
6	Rosiglitazone	Inferred	79
7	Furazolidone	Inferred	7
8	Hydrocortisone	Inferred	30
****9****	****Isocarboxazid****	****NA****	****NA****
****10****	****Ajmaline****	****NA****	****NA****
11	1-(5-Isoquinolinesulfonyl)-2-Methylpiperazine	Inferred	15
12	Troglitazone	T	86

Evidence represents a drug–disease association is curated, inferred, or does not exist in CTD. Curated associations include three types: marker/mechanism (Evidence = "M"), therapeutic (Evidence = "T"), and marker/mechanism & therapeutic (Evidence = "M&T"). If an association is inferred by CTD, Evidence = "inferred", and if it does not exist in CTD, Evidence = "NA"; Ref Count represents the number of reference (s) for the curated and inferred associations. If an association does not exist in CTD, the Ref Count = NA.

### Analysis of potentially HCC-related drugs based on CMap database

The CMap database not only can be applied to calculate drug–disease correlations but also can be used to identify connections between two drugs. In particular, for the same disease, if two drugs have a strong positive relationship, they may have similar effects on this disease. On the contrary, if their relationship is negative, they may have opposite effects. In this section, based on CMap, we further analyzed the three therapeutic drugs: securinine, isocarboxazid, and ajmaline. From CMap, we obtained the gene expression data related to eight HCC drugs, which were marked as “therapeutic” in the CTD database: daunorubicin, troglitazone, paclitaxel, doxorubicin, sirolimus, nimesulide, dexamethasone, and genistein. We estimated their correlations with six predicted drugs using formula (6) (see the details in Methods section) based on their related gene differential expression data. For the eight therapeutic drugs of HCC, troglitazone is predicted based on our method as indicated in Table 3. The other 7 drugs were not found in the top 30 initial prediction. A more likely reason is that they target other mechanisms that are not covered by the pathways predicted from the HCC network or have mechanisms that act besides gene expression.

For securinine, it yields a highly positive therapeutic score with most of the eight drugs shown in Fig 4B. We took daunorubicin, troglitazone, and paclitaxel as examples to analyze. Daunorubicin is a chemotherapy medication used to treat cancer. The liposomal formulation of the anthracycline daunorubicin has low systemic toxicity and is taken up strongly by the liver. Dianele et al. [56] have found that daunorubicin was an active agent against HCC. Troglitazone is a thiazolidinedione Peroxisome proliferator-activated receptor gamma (PPARγ) agonist that exhibits antidiabetic, anticancer, antifibrotic, and anti-inflammatory activities. This drug was withdrawn from the US market in 2000 due to high hepatotoxicity [57]. Yu et al. [58] have found a significant decrease in expression of PPARγ mRNA and protein in human liver cancers compared with surrounding non-tumorous liver. Troglitazone induces PPARγ expression and inhibits cell growth both *in vitro* and *in vivo* [59]. Paclitaxel is a cancer medication that interferes with the growth and spread of cancer cells in the body [60]. Gagandeep et al. [61] have indicated that paclitaxel is cytotoxic to cultured HCC cells, and now paclitaxel is a common anticancer agent for therapy of HCC patients [62–64]. Ajmaline receives high positive scores with nimesulide, dexamethasone, doxorubicin, and paclitaxel. Nimesulide is a cyclooxygenase-2 (COX-2) inhibitor and is used to inhibit the proliferation and promote apoptosis of HepG2 by upregulating SMAD Family Member 4 (SMAD4) [65]. Dexamethasone, a type of corticosteroid medication, can restore gluconeogenesis in malignant hepatocytes by bypassing the abnormal regulation of 11β-Hydroxysteroid dehydrogenase (11β-HSD) enzymes, which indicates that targeting altered metabolism in liver cancer could prove useful as a therapeutic strategy for HCC [66].

Paclitaxel is one of the therapeutic drugs of HCC (labeled as “therapeutic” in CTD). We also calculated the correlation between 1309 drugs in CMap and paclitaxel and ranked all of them in descending order. The rankings of the three potential HCC drugs (securinine, isocarboxazid and ajmaline) we predicted among the 1309 drugs are: 69, 845 and 1226. The drug ajmaline in the existing verification aspects (see Table 3 and Figs 3B and 4B) all indicated that it is likely to be a potential drug for the treatment of HCC. Therefore, predicting potential therapeutic drugs based only on gene expression data under the action of drugs is not comprehensive enough. It is necessary to consider the tissue specificity and functional pathway information of the disease, which shows the rationality of our algorithm.

These results indicated that our method can be used to identify therapeutic drugs for diseases. Moreover, our method can be applied to mark the known drug–disease associations as positive or negative, such as some drug–disease associations in CTD database, to provide a better basis for the treatment of diseases.

### Analysis of potentially HCC-related drugs based on of pathway enrichment

Furthermore, we tried to apply the DAVID functional annotation tool [43] to identify enriched KEGG pathways for each of the six drugs based on their targets. If the targets of a drug had a close correlation with HCC-related pathways, the drug likely would be helpful for the treatment of HCC.

Isocarboxazid has two targets “MAOA” and “MAOB”, and they are related to 13 KEGG pathways. Some of these targets have close relationships with liver cirrhosis or HCC. For example, for the alcoholism pathway, Mckillop et al. [67] have found that chronic alcohol consumption had long been associated with progressive liver disease toward the development of hepatic cirrhosis and the subsequent increased risk for developing HCC. Metabolic pathways are linked to a series of chemical reactions occurring within a cell. Huang et al. [68] have shown that the metabolism in the HCC tumor was modified to promote cellular proliferation or to escape from apoptosis. For the drug metabolism–cytochrome P450 pathway, cytochrome P450 1A1 is a major enzyme in the bioactivation of exogenous procarcinogens of HCC [69]. The drug ajmaline has one enriched pathway, adrenergic signaling in cardiomyocytes. The adrenergic signaling pathway plays an important role in cancer progression by regulating multiple cellular processes such as angiogenesis, invasion and apoptosis [70–75].

These results further showed that these potential drugs have close relationships with HCC. With the improvement of data, our algorithm will perform even better.

### Experimental validation of potentially HCC-related drugs via *in vitro* cellular assay

**Fig 5:**
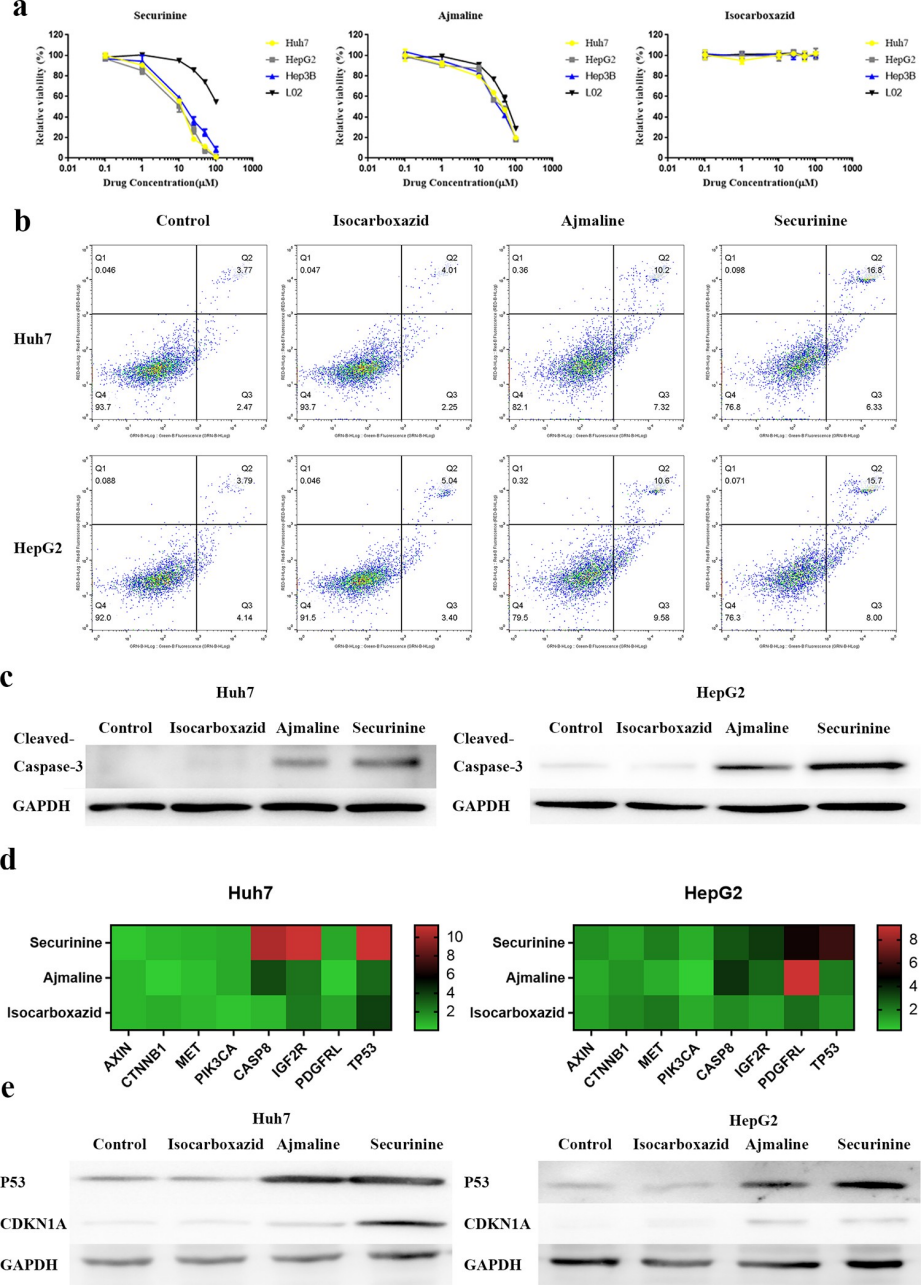
Experimental validation of the predicted drugs via *in vitro* cellular assays. **(a)** Experimental validation of predicted anticancer effects of drugs in the cell viability. HCC cell lines Huh7, HepG2 and Hep3B, and human normal liver cell line L02 were used in the experiment. Both cell lines were treated with 0, 0.1, 1, 10, 25, 50, and 100 μM different drugs for 72 h. **(b)** The effect of therapeutic drugs to elevate cell apoptosis ratio. Cells were treated with 15 μM different drugs for 72 h. **(c)** Western blot detection for drug-induced Caspase-3 cleavage. Both cell lines were treated with 15 μM different drugs for 72 h. No-treatment sample as control. **(d)** Quantification of the ratio of changes in HCC-related genes induced by selected drugs. **(e)** Western blot analysis of P53 and CDKN1A in the indicated cells upon 72 h by treatment with 15 μM selected drugs. All experiments were performed three times.

We used HCC cell lines Huh7, Hep3B, and HepG2 cells to detect the inhibitory effects of drugs on HCC cell viability, and the human noncancerous liver cell line L02 was used as the normal cell control. Based on our experimental results, we found that securinine and ajmaline significantly inhibited cell viability of HCC cells (Fig 5A). However, the two drugs have different ability to inhibit cell activity. Intriguingly, securinine inhibits the activity of HCC cell lines at low concentrations (IC50: Huh7, 12.81±1.13 μM; HepG2, 13.95±1.12 μM; Hep3B, 16.8±1.13 μM) and has a weaker ability to inhibit L02 normal cell lines (IC50: L02, 161.59±0.7 μM). The inhibitory effect of ajmaline on the activity of normal cell lines is also weaker than that of HCC cell lines (IC50: Huh7, 38.26±0.45μM; HepG2, 37.98±0.44 μM; Hep3B, 35.41±0.15 μM; L02, 57.03±0.54 μM), but the IC50 between normal cell lines and HCC cell lines is relatively close, which indicates that the use of ajmaline *in vivo* may have relatively high liver toxicity. In the HCC cell line, the IC50 value of securinine treatment for 72h is about 15μM. Thus, we used the same drug concentration (15 μM) for the follow-up drug experiments in Huh7 and HepG2 cells. In the assay of cell apoptosis, securinine and ajmaline significantly increased the apoptosis ratio compared with control, whereas isocarboxazid had no significant effect (Fig 5B). We also tested Caspase-3 cleavage via western blot assay in Huh7 and HepG2 cells (Fig 5C). In the dosing process, securinine and ajmaline significantly induced the level of Caspase-3 cleavage, which is the marker of apoptosis, and securinine showed a stronger effect than ajmaline.

Due to the effects of some of the selected drugs on tumor cells were less than expected, we performed qPCR assay to verify the effects of drugs on the expression of HCC-associated genes identified with OMIM (Fig 5D). We found that the effective HCC therapeutic drugs, securinine and ajmaline, showed a significant effect on the expression of tumor suppressor genes in Huh7 and HepG2 cells.

As an important tumor suppressor gene, the mRNA level of TP53 was positively regulated by therapeutic drugs. We further verified the regulatory effects of different drugs on the expression of TP53 pathway-related proteins by western-blot (Fig 5E). We found that securinine and ajmaline can upregulate the protein levels of TP53 as well as its downstream protein CDKN1A.

### Performance comparison with other algorithms

**Fig 6:**
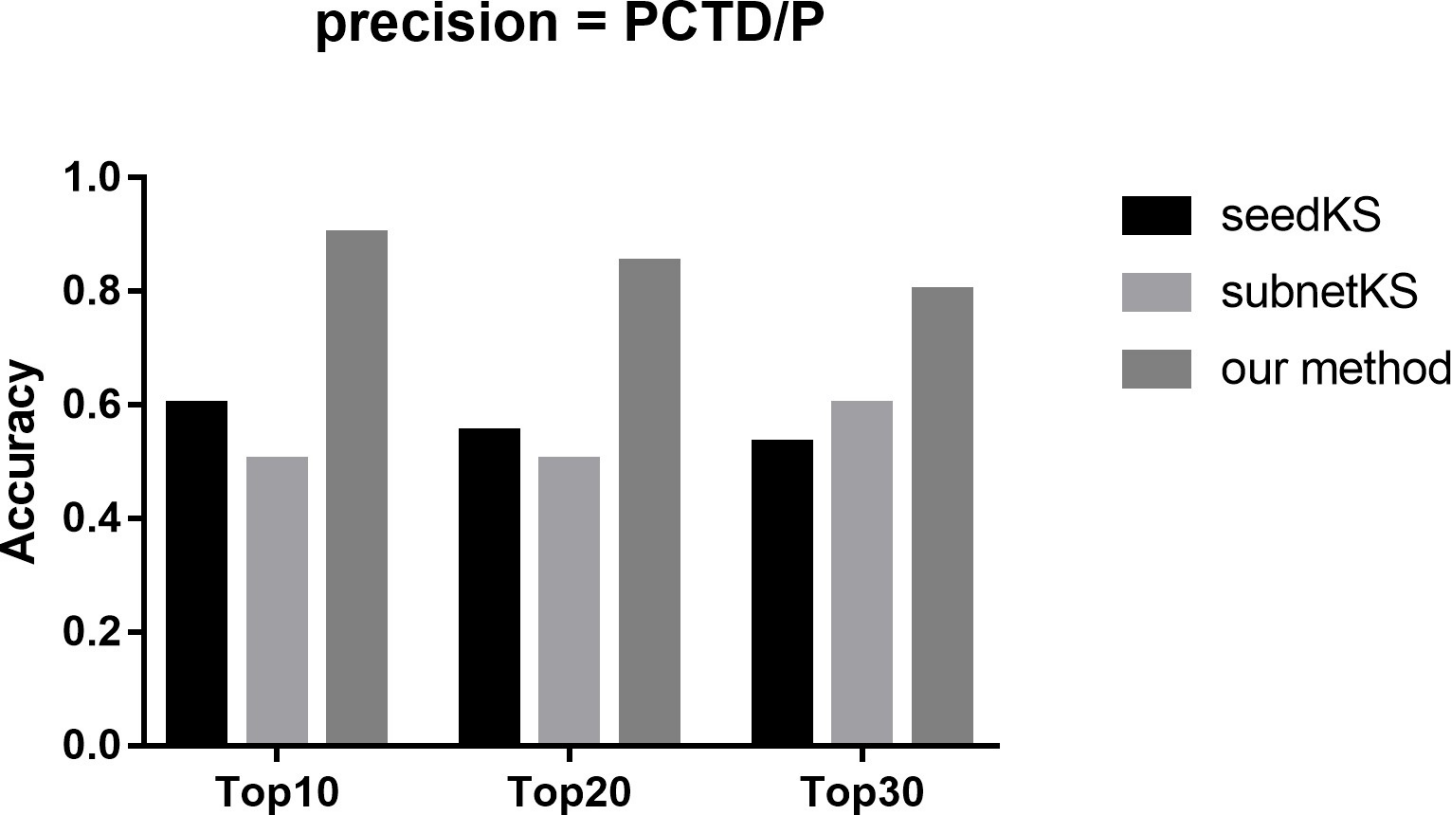
Performance comparison with other algorithms on top prediction.

To further illustrate the effectiveness of our algorithm, we compared our method with other simpler approaches that used a subset of the used experimental and prior knowledge data: a) seedKS: Identify drugs that reverse cancer associated gene expression signatures using their rank-based method (ignore pathways and network). b) subnetKS: Identify drugs that reverse the signatures of those 57 genes that are part of the liver cancer network using their rank-based method (consider network, ignore the pathways). c) seedPathKS: Predict pathways from the DEGs (Differentially Expressed Genes) (ignore the cancer network) and find drugs that reverse gene expression signatures in these pathways. d) our method: Use the method as described in the paper. Through analysis, we found that the algorithm seedPathKS, which is based on seed nodes for pathway enrichment (count ≥5 and p-value ≤0.001 (consistent with the article)), has not enriched any pathway, and therefore cannot predict potential drugs. Therefore, there is no result of the algorithm seedPathKS in Fig 6. In Fig 6, we compared the results of three algorithms: seedKS, subnetKS and our method. Based on the benchmark database CTD, the precision (see formula (1)) of top10, top20, and top30 predicted by the three algorithms are compared respectively. In all the three cases, our method has the highest precision as shown in Fig 6. Furthermore, for the three drugs newly predicted by our algorithm: Securinine, Isocarboxazid and Ajmaline (see Table 3), none of them appeared in the top 30 prediction results of the algorithms seedKS and subnetKS.

## Discussion

HCC, also called malignant hepatoma, is the most common type of liver cancer with poor prognosis. It is still necessary to develop more effective and affordable treatment options for HCC. Drug repositioning represents a profitable strategy to accelerate drug discovery. In this study, we proposed an approach to identify potential drugs for the treatment of HCC that considered the special relationships among HCC, liver tissue, and KEGG pathways. This study clearly distinguished therapeutic drugs for HCC, which made it possible to provide a more accurate reference for the treatment of HCC. Finally, we chose the top-three therapeutic drugs potentially related to the treatment of HCC for further analysis.

In our experimental results, two of the three selected therapeutic drugs, securinine and ajmaline, did cause a significant inhibition of HCC cells. We found that securinine had a significant toxicity effect on cancer cells. Securinine is widely used in the treatment of polio sequelae and facial nerve paralysis [76,77], which is safer than than strychnine with 26-fold higher LD50 in rats following intravenous administration [78]. At the same time, it has also been verified that securinine is safety for *in vivo* experiments. By intravenous injections, LD50 of securinine nitrate in mice was 6.23 ± 0.16 mg/kg, whereas 15.1±0.48 mg/kg in rats [79]. As a potential anti-tumor agent, existing evidence shows that securinine has a significant inhibitory effect on various tumor cell lines at a concentration of 20 μM [80]. We found that securinine had a significant toxicity effect on cancer cells. Securinine is considered to have significant effects on the treatment of a variety of tumors, which indicates that same effect of securinine in the liver cancer appears to be certifiable [81,82]. Because securinine is metabolized primarily in the liver, this may result in a higher concentration of securinine in the liver than in other tissues [83]. Therefore, we believe that securinine may have a strong cytostatic effect on HCC, especially compared with other cancer cells, as it has a much weaker effect on normal cells. The suppression effect of ajmaline on cell viability was lower than securinine, and ajmaline had a similar inhibiting effect on normal cells. The change of potassium ion level affects the apoptosis of HCC cells [84]. Ajmaline can alter the intracellular K+ ion concentration by blocking the cell Na+/K+ channels, and it has been proved to be cytotoxic to tumor cells in our experiments, which proves that ajmaline has potential therapeutic value, although it has similar cytotoxicity to normal liver cells [85]. In fact, in a culture environment, some excellent targeted therapeutic drugs, such as sorafenib, will also have similar cytotoxicity to normal cell lines [86]. This may be due to the drugs inhibiting the corresponding regulatory pathways and leading to undifferentiated cell death, regardless of whether the cell line is a normal cell line or a cancer cell line. The occurrence of HCC is often accompanied by gene mutations, and HCC driven by different gene mutations should have different treatment options. Ajmaline may have potential therapeutic value for HCC with Na+/K+ channel gene mutations. We also did not find an inhibitory effect of isocarboxazid, which indicated that isocarboxazid may not have an inhibitory effect on HCC. The use of MAO inhibitor alone seemed to have no effect on HCC cells [87]. Isocarboxazid may be used as an adjuvant drug, concurrently with chemotherapy drugs, but this was not verified in our experiment. It should be noted that we have not conducted *in vivo* experiments to verify the hepatotoxicity of the drug, and *in vitro* experiments cannot perfectly verify the effectiveness of the drug *in vivo*, thus the results we obtained need to be further proved in follow-up research.

We experimentally validated drug-regulated gene expression of therapeutic drugs. The results showed that drugs with cytotoxic effects on HCC cells positively regulated the expression of tumor suppressor genes in the eight genes we selected and promoted the regulation of downstream genes by tumor suppressor genes. These results indicated that our identification of HCC-related genes and methods for predicting drugs by HCC-specific pathways were correct and feasible.

We confirmed that two of the three newly predicted therapeutic drugs had anticancer effects. Securinine was extremely interesting because this drug affected only HCC cell viability but had little cytostatic effect on normal cells. Because our proposed method was aimed at known drugs, and some of the drugs lacked published research, the predicting efficiency could not be fully guaranteed. Beyond our experimentally tested drugs, however, a variety of other drugs, such as geldanamycin and clozapine [81,88], with potential therapeutic significance for HCC have been proven to have good therapeutic results in HCC *in vivo* or *in vitro*. All these findings indicated that our method was meaningful and worthy of further development and it may be used as an integrative tool to identify HCC-specific drugs from among approved drugs. In summary, we proposed a new approach to identify therapeutic drugs for HCC from existing drugs, by investigating functional information of pathways, changes in pathways and individual genes, and special relationship between HCC and liver tissues. Our method aims at identifying drug candidates that have a high likelihood to inhibit HCC growth for experimental testing, rather than finding all drugs that inhibit HCC growth. The latter might also be complicated, since there possibly are multiple different mechanisms that can be successfully targeted, while our approach only addresses one of them. The drugs obtained by our proposed method may have clinical value, which requires further validation by experiments. With the continuous improvement of data, such as integrating additional pathway information and tissue data, our proposed method will more accurately predict novel drugs for diseases. Our proposed method considered the special relationship between tumors, related tissues, and KEGG pathways. It may have similar meanings for tumors in other tissues. Therefore, the method of data analysis in this study could also be applied to predict the potential drugs for other types of tumors by changing the database.

## Materials and methods

### Normalization of the expression values for all genes

We used the following formula [89] to standardize the expression values of all genes:

$$z_{ij}=\frac{g_{ij}-mean(g_{i})}{std(g_{i})}$$ (2)

Where *g*_*ij*_ represents the expression value of gene *i* in sample *j*; and *mean*(*g*_*i*_) and *std*(*g*_*i*_), respectively, represent the mean and standard deviation of the expression vector for gene *i* across all samples.

### Calculation of the therapeutic scores

Based on the gene expression profile under the action of drugs and the collection of characteristic pathways of HCC, we inspired by the Kolmogorov-Smirnov test [24,45] and developed the following approach:. For the cancer DEGs (Differentially Expressed Genes) and the CMap drug DEGs, we firstly ranked them in descending order according to their log2 (fold changes) respectively, with low number ranks being assigned to the most upregulated genes and high number ranks being assigned to the most downregulated genes. Secondly, the cancer DEGs were divided into two sets: up-regulated gene set and down-regulated gene set. For the cancer up-regulated gene set, for example, we got the intersection of these genes and all the drug DEGs, that is, *m* common genes. Thirdly, for these *m* genes, we re-ranked them in descending order according to their differential expression values under the action of drugs from large to small and marked them as *G*_*up*_. Similarly, the reordered cancer down-regulated gene set *G*_*down*_ can be obtained. Finally, based on the *m* genes in *G*_*up*_ or *G*_*down*_, we calculated the correlations between drugs and diseases.

For an HCC pathway *i* and a drug expression profile *j*, we computed a therapeutic score separately for the set of up- or downregulated genes in the pathway *i*: $CS_{up}^{i,j}$ or $CS_{down}^{i,j}$. For the HCC pathway *i*, it needs up- (*G*_*up*_) or downregulated genes (*G*_*down*_) as inputs. The computational formulas are as follows [24]:

$$a=\overset{m}{\underset{p=1}{Max}}\left[\frac{p}{m}-\frac{V\left(p\right)}{n}\right]$$ (3)

$$b=\overset{m}{\underset{p=1}{Max}}\left[\frac{V\left(p\right)}{n}-\frac{p-1}{m}\right]$$ (4)

$$CS=\left\{ \begin{array}{l} a(if\;a>b)\\ -b(if\;a<b) \end{array}\right.$$ (5)

where *n* represents the total number of genes in the reference drug expression profile *j*; *m* represents the number of upregulated genes *G*_*up*_, or the number of downregulated genes *G*_*down*_; *p* represents the position of the input set (*p* = 1…*m*); *V*(*p*) is the position of the *p*th input gene in the gene list of drug expression profile *j*; and *CS* represents the connectivity score $CS_{up}^{i,j}$ or $CS_{down}^{i,j}$ for the up- or downregulated gene set.

Taking the up-regulated gene set *G*_*up*_ as an example, we expect the genes in *G*_*up*_ to be at the back of the drug DEG list, so that the drug has the potential to treat cancer. That is, for a gene with a small *p* value in *G*_*up*_, we expect its *V*(*p*) to be large. At this time, the value of *b* is greater than *a*, and *CS* takes −*b*. Taking the maximum value ensures that *V*(*p*) is located at the bottom of the drug DEG list as much as possible, and *p* is located at the top of *G*_*up*_. In this way, other genes after the *p* position in *G*_*up*_ will be distributed after the *V*(*p*) position in the drug DEG list, ensuring that most of the up-regulated genes of the disease are distributed at the bottom of the drug DEG list. Therefore, we considered all or most of the genes in each pathway. For genes in *G*_*down*_, we expect these genes to be at the top of the drug DEG list, so that the drug has the potential to treat cancer. That is, for a gene with a large *p* value in *G*_*down*_, we expect its *V*(*p*) to be small. At this time, the value of *a* is greater than *b*, and *CS* takes *a*. Similarly, taking the maximum value ensures that the genes before the *p* position in *G*_*down*_ are all before the *V*(*p*) position in the drug DEG list. In this way, all or most of the down-regulated genes of cancer are distributed in the upper part of the drug DEG list. That is, the drug has the potential to treat cancer.

The therapeutic score (*TS*) between a drug with *k* instances in CMap and HCC with 12 functional pathways is calculated as follows [45]:

$$TS=\frac{1}{k}\sum_{i=1}^{12}\sum_{j=1}^{k}CS^{i,j}$$ (6)

where the drug has *k* instances in CMap and HCC has 12 functional pathways; and *CS*^*i*,*j*^ represents the connectivity score between HCC pathway *i* and instance *j*, which is defined as follows:

$$CS^{i,j}=CS_{up}^{i,j}-CS_{down}^{i,j}$$ (7)

where $CS_{up}^{i,j}$ and $CS_{down}^{i,j}$ represent the connectivity scores for the upregulated gene set and the downregulated gene of pathway *i*, respectively, and their definitions are given in formulas (2) to (4). That is, there are 12 pathways related to HCC and a drug has *k* instances. We calculate the KS correlation between each of the 12 pathways and each of the *k* instances, and we can get (12×*k*) KS values. We add them up and finally the sum is divided by *k* to find the average KS value as the correlation between the drug and HCC.

### Chemicals

The securinine was obtained from Beijing Solarbio Science and Technology (Beijing, China). Isocarboxazid and ajmaline were obtained from Santa Cruz Biotechnology (Santa Cruz, Dallas, TX, USA). The purity of these chemicals was ≥98% according to the instructions.

### Cells and cell culture

Human HCC cell lines, HepG2, Hep3B and Huh7 were obtained from Procell Life Science and Technology Co., Ltd. (Wuhan, China), and L02 cells were purchased from the Shanghai Institute of Biochemistry and Cell Biology, Chinese Academy of Sciences (Shanghai, China). Cells were cultured in Dulbecco’s Modified Eagle’s Medium (DMEM; M&C Gene Technology Co., Ltd., Beijing, China) and supplemented with 10% (v/v) fetal bovine serum (Gibco Life Technologies, Waltham, MA, USA), 100 U/mL of penicillin, and 100 U/mL of streptomycin. Both cells were incubated in a humidified 5% (v/v) CO_2_ at 37°C incubator.

### Cell viability analysis

For the cell viability, cells were seeded into 96-well plates at a density of 2.5 × 10^3^ cells per well. Then, cells were treated with different concentrations of drugs. Four sets of cells were treated with different solvents as a control. After cells were cultured for 72 h, we added 10 μL of CCK-8 (CCK-8 Cell Proliferation and Cytotoxicity Assay Kit; Bioworld Technology, Nanjing, China) to each well followed by incubation for 4 h at 37°C. Cell viability was assessed by detection of absorbance at 450 nm using a spectrophotometer (Bio-Rad, Hercules, CA, USA). The experiment was repeated three times.

### Flow cytometric analysis for apoptosis

Cells were treated with different drugs at 15 μM for 72 h. Cells were harvested by centrifugation, washed twice with cold phosphate buffered saline (PBS), and then analyzed by BD FACSCanto II flow cytometry (BD Biosciences, Franklin Lakes, NJ, USA) according to the protocol of the Annexin V-FITC/PI Apoptosis Detection Kit (Bioworld Technology). We used FlowJo Software (version 10; FlowJo LLC, Ashland, OR, USA) to analyze these data.

### Western blot analysis

Before protein extraction, cells were washed by 1 × PBS to remove the culture media. Cells were collected and lysed with BC-200 lysis buffer (20 mM HEPES buffer, pH 7.9, containing 200 mM KCl, 1 mM EDTA, 10 mM β-mercaptoethanol, 0.2 mM PMSF, 0.1% NP-40, and 10% glycerol). Protein samples were separated by 10% sodium dodecyl sulfate–polyacrylamide gel electrophoresis and were transferred to polyvinylidene fluoride membrane (Millipore, Bedford, MA, USA). After blocking with 5% nonfat milk at room temperature for 1 h, the membranes were probed with primary antibodies at 4°C overnight. Then, after four times 1 × TBS with Tween-20 wash, we incubated the membranes with a corresponding secondary antibody at room temperature for 1 h. The primary antibodies used in this study included P53 and CDKN1A (Santa Cruz, Dallas, TX, USA), Caspase3 and cleaved-Capase3 (Abcam, Cambridge, UK), GAPDH (Proteintech, Wuhan, China), and the secondary antibodies includes goat anti-rabbit antibody and goat anti-mouse antibody. The secondary antibodies were obtained from Jackson ImmunoResearch Inc. (West Grove, PA, USA). Bound antibodies were visualized using an enhanced chemiluminescence kit (Wanleibio, Dalian, China)

### RNA extraction, reverse transcription, and real-time PCR

RNAiso Plus (Takara, Shiga, Japan) was used to extract the total RNA and evaluated the concentration and quality of RNA using NanoDrop 2000 Spectrophotometer (Thermo Fischer Scientific, Waltham, MA, USA). Complementary DNA was generated by using the RevertAid First Strand cDNA Synthesis Kit (Thermo Fisher Scientific) according to the protocol. The primer sequences for detecting genes were listed in S1 Table.

We performed real-time PCR on the CFX96 Touch System (Bio-Rad) using THUNDERBIRD SYBR qPCR Mix (Toyobo, Osaka, Japan). The expression of genes was calculated using the 2^-ΔΔCt^ method with GAPDH as the reference gene.

### Statistical analysis

All data were analyzed using Graph Pad Prism software (San Diego, CA, USA). *P*-value ≤0.05 was used as a statistically significant test.

## Supporting information

S1 Table Primers used in the present study for detecting mRNA levels of genes. (XLSX)

S2 Table Liver-specific subnetwork extended by eight related genes of HCC. (XLSX)

S3 Table Twelve HCC tissue-specific pathway signatures. (XLSX)

S4 Table A total of 1,168 ranked drugs in descending order by their relationships with HCC. (XLSX)
